# *Glycyrrhiza uralensis* water extract enhances dendritic cell maturation and antitumor efficacy of HPV dendritic cell-based vaccine

**DOI:** 10.1038/srep43796

**Published:** 2017-03-08

**Authors:** Adila Aipire, Jinyu Li, Pengfei Yuan, Jiang He, Yelang Hu, Lu Liu, Xiaoli Feng, Yijie Li, Fuchun Zhang, Jianhua Yang, Jinyao Li

**Affiliations:** 1 Xinjiang Key Laboratory of Biological Resources and Genetic Engineering, College of Life Science and Technology, Xinjiang University, Urumqi 830046, China; 2 Key laboratory of Xinjiang Uighur Medicine, Xinjiang Institute of Materia Medica, 140 Xinhua South Road, Urumqi 830004, China; 3 XinJiang DingJu Biotech CO., LTD, 181 Xicai Road, Urumqi 830000, China; 4 Texas Children’s Cancer Center, Department of Pediatrics, Dan L. Duncan Cancer Center, Baylor College of Medicine, TX 77030, USA

## Abstract

Licorice has been used as herbal medicine and natural sweetener. Here, we prepared *Glycyrrhiza uralensis* water extract (GUWE) and investigated the effect of GUWE on the maturation and function of dendritic cells (DCs) and its adjuvant effect on DC-based vaccine. We observed that GUWE dose-dependently promoted DC maturation and cytokine secretion through TLR4 signaling pathway. The capacity of DC to stimulate allogenic splenocyte proliferation was also enhanced by GUWE treatment. Compared with control group, GUWE treated DCs pulsed with human papillomavirus (HPV)-16 E6/E7 peptides significantly inhibited the tumor growth in both early and late therapeutic groups. In early therapeutic group, the frequencies of induced regulatory T cells (iTregs: CD4^+^CD25^−^Fopx3^+^) and CD4^+^ and CD8^+^ T cells were significantly decreased and increased, respectively. HPV-16-specific CD8^+^ T cell responses were significantly induced and negatively correlated with iTreg frequencies and tumor weight. These results indicated the immunoregulatory activities of licorice.

Persistent infection of human papillomavirus (HPV) is an essentially causal factor for the pathogenesis of cervical cancer, especially high-risk types of HPV, and may also caused other types of cancers including penis, vulva, vagina, anus and oropharynx^1^,^2^,^3^,^4^. Although two prophylactic HPV vaccines (quadrivalent Gardasil^®^ and bivalent Cervarix^TM^) are commercially available^5^,^6^, the HPV infections and its related diseases remain the global and regional burden^7^. Approximately 86% of the infections and 88% of the deaths are from the developing countries due to the lack of the screening programs or prophylactic HPV vaccines^8^. The two prophylactic vaccines cannot eliminate the established HPV infections. Therefore, several types of therapeutic HPV vaccines have been investigated such as live vector vaccines, nucleic acid vaccines, peptide/protein vaccines and dendritic cell (DC)-based vaccines^3^,^9^.

DCs are professional antigen presenting cells and play a pivotal role in the induction of antigen-specific immune responses. Upon contacting with ‘danger signal’, immature DCs undergo the process of maturation to become mature DCs that provide different signals such as MHC-peptide complex, co-stimulatory molecules and cytokines to naïve T cells to promote the differentiation of T helper (Th) cells^10^. However, accumulating evidence showed that tumors could induce immunosuppressive microenvironment, such as dampening the maturation and function of DCs^11^,^12^. To overcome the drawbacks of DCs in cancer patients, efforts were focused on the *ex vivo* manipulated DCs, which is used in around 97% of the clinical trials^13^. It has been well documented that DC-based vaccines are safe and can generate antigen-specific immune responses^13^,^14^. However, DC-based vaccines are not very successful in clinical trails due to suboptimal DC maturation with low secretion of IL-12, poor migration and decreased survival^14^,^15^,^16^,^17^. IL-12 directs the generation of Th1 responses and cytotoxic T lymphocytes (CTL)^18^,^19^. In order to improve the efficacy of DC-based vaccines, the *ex vivo*-differentiated DCs need to be stimulated by adjuvant to become the fully mature DCs with high level of IL-12 secretion. However, the availability of clinical grade adjuvants including TLR agonists is limit^20^,^21^.

It has been demonstrated that herbal medicine and its components have the immunomodulatory effects, especially for the maturation of DCs^22^,^23^. Licorice has been extensively used as herbal medicine and natural sweetener in various kinds of foods, which contains many components including triterpene saponins, flavonoids, isoflavonoids and polysaccharides, and has various pharmacological effects such as antiinflammation, antioxidation, antivirus, antimicrobe, antitumor and immunoregulation^24^,^25^,^26^,^27^. Here, we explored the effects of *Glycyrrhiza uralensis* water extract (GUWE) on the maturation and function of DCs, and evaluated the antitumor efficacy of HPV-16 E6/E7 peptides pulsed DCs stimulated with GUWE in TC-1 tumor mouse model.

## Results

### The qualitative analysis of GUWE by LC-MS/MS

GUWE was prepared and the quality control was done by LC-MS/MS through comparing with the standard glycyrrhizin. The negative mode ESI-MS/MS spectra of glycyrrhizin and GUWE were obtained. Standard glycyrrhizin (retention time: t_R_ = 8.18 min) showed a [M–H]^−^ ion at m/z 821.3951 (Supplementary Fig. S1A and B), which is consistent with its molecular weight. Using the same conditions, we got the total ion chromatogram of GUWE (Supplementary Fig. S1C). The components with m/z 821.4 were further analyzed according to their retention times and found 5 peaks (Supplementary Fig. S1D). One peak (t_R_ = 8.2 min) displayed [M–H]^−^ ion at m/z 821.3974 (Supplementary Fig. S1E), which is same as glycyrrhizin. The results suggested that GUWE contained glycyrrhizin.

### GUWE promotes the maturation of DCs

**Figure 1:**
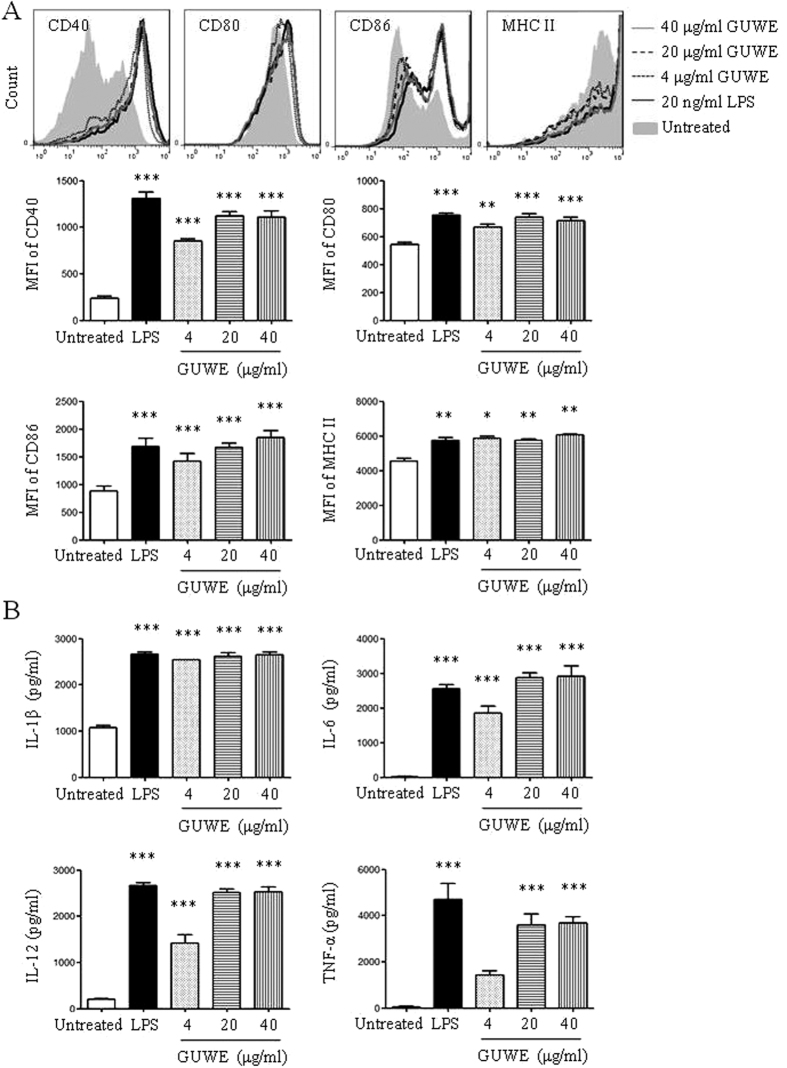
DC maturation and cytokine production upon GUWE treatment. DCs were induced from bone marrow of C57BL/6 mice in the presence of GM-CSF. On day 7, cells (1 × 10^6^/ml) were treated with different concentrations (4, 20 and 40 μg/ml) of GUWE for 12 h. LPS (20 ng/ml) was used as positive control. (**A**) After treatment, the expressions of co-stimulatory molecules and MHC II on DCs were detected by flow cytometry (upper panels). The mean fluorescence intensity (MFI) (mean ± SEM) of co-stimulatory molecules and MHC II is shown in lower panels. (**B**) The supernatants was collected and the production of IL-1β, IL-6, IL-12 and TNF-α was detected by ELISA. The concentrations (mean ± SEM) of cytokines are shown. Data are from 4 independent experiments and analyzed by ANOVA. *p < 0.05; **p < 0.01; ***p < 0.001 compared to untreated DCs.

10 mg/ml of GUWE was prepared and the concentration of polysaccharide was measured, which is 2.5 mg/ml. To avoid the effect of endotoxin in GUWE on DC maturation, the level of endotoxin was detected by Gel Clot TAL assay. The result showed that the level of endotoxin in GUWE is undetectable (Supplementary Fig. S2). Then, different concentrations (4, 20 and 40 μg/ml) of GUWE were used to treat DCs to detect the effect of GUWE on DC maturation. LPS was used as positive control. After 12 h, the apoptosis and necrosis of DCs were detected by Annexin V/PI staining. The expressions of CD40, CD80, CD86 and MHC II on DCs were tested by flow cytometry. The supernatant was collected to detect the production of IL-1β, IL-6, IL-12 and TNF-α by ELISA. The results of Annexin V/PI staining showed that GUWE did not induce the apoptosis and necrosis of DCs (Supplementary Fig. S3), suggesting that these doses are safe for DCs *in vitro*. We observed that GUWE significantly upregulated the expressions of CD40, CD80, CD86 and MHC II (Fig. 1A), and increased the concentrations of IL-1β, IL-6, IL-12 and TNF-α (Fig. 1B), in a dose-dependent manner. Moreover, the endocytosis of DCs was evaluated by FITC-dextran. Untreated, GUWE or LPS treated DCs were incubated with FITC-dextran for 1 h and the cells were analyzed by flow cytometry. Compared to untreated DCs, the frequencies of FITC^+^ DCs were significantly decreased by GUWE or LPS treatment (Supplementary Fig. S4). These results suggest that GUWE promotes the maturation of DCs.

### GUWE enhances the function of DCs

**Figure 2:**
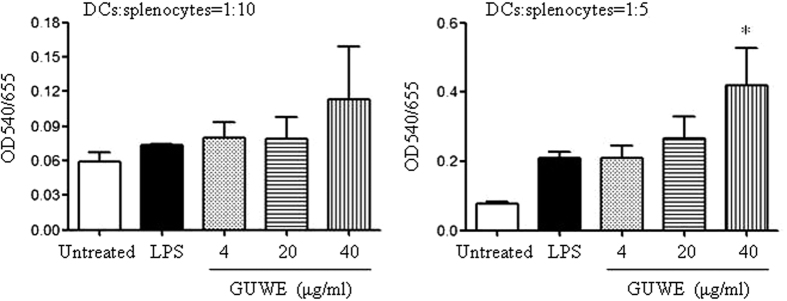
The function of DCs upon GUWE treatment. MLR was performed using C57BL/6 DCs and BALB/c splenocytes. DCs on day 7 were treated with different concentrations (4, 20 and 40 μg/ml) of GUWE or LPS for 12 h, and then treated with mitomycin C. Splenocytes were obtained from BALB/c mice. DCs and splenocytes at ratios of 1:5 and 1:10 were co-cultured for 48 h. Cell proliferation was detected by MTT assay. Data are from 3 independent experiments and analyzed by ANOVA. *p < 0.05 compared to untreated DCs.

Next, we detected the effect of GUWE on the function of DCs by mixed lymphocyte reaction (MLR). DCs were induced from bone marrow of C57BL/6 mice and treated with different concentrations of GUWE or LPS, which were co-cultured with splenocytes from BALB/c mice at different ratios. After 72 h, the proliferation of splenocytes was detected by MTT assay. The results showed that DCs treated with 40 μg/ml of GUWE significantly enhanced the proliferation of splenocytes at the ratio of DCs:splenocytes = 1:5 (Fig. 2), suggesting that GUWE enhances the function of DCs.

### GUWE promotes DC maturation via TLR4 signaling pathway

**Figure 3:**
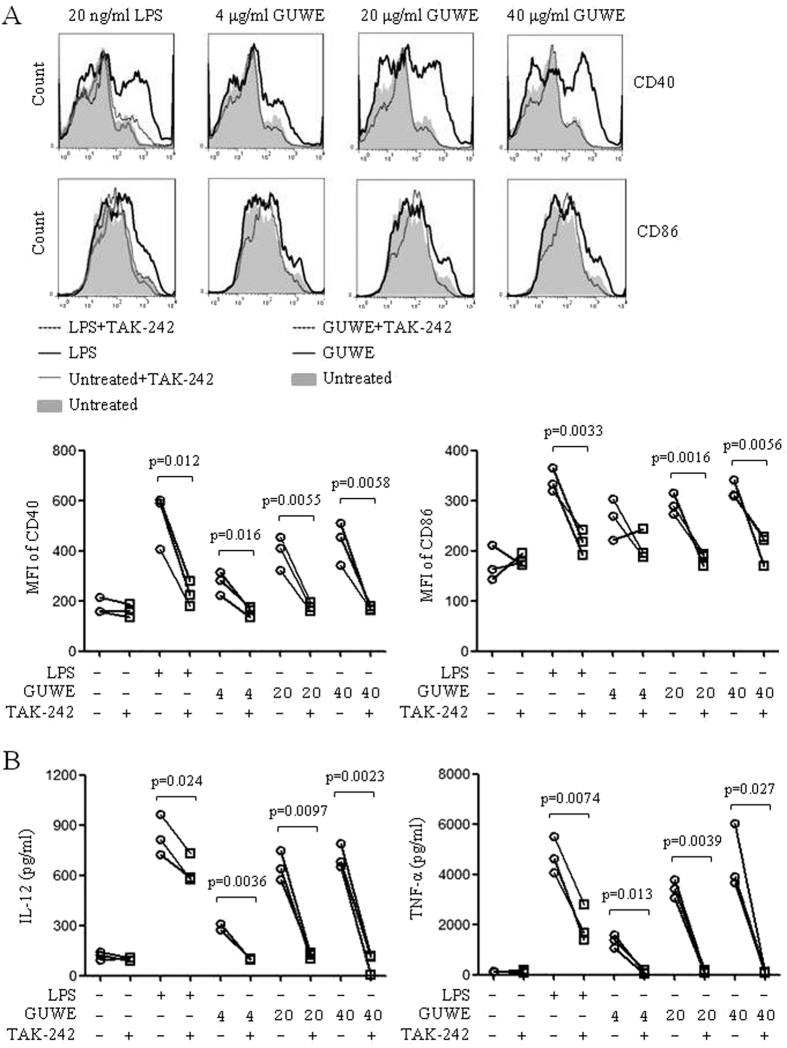
The effect of TLR4 inhibitor on DC maturation and cytokine production. DCs were pretreated with or without 1 μM TAK-242 for 1 h, and then treated with 4, 20 and 40 μg/ml of GUWE or 20 ng/ml of LPS for 12 h. (**A**) The expressions of CD40 and CD86 were analyzed by flow cytometry. MFI of CD40 and CD86 are shown. (**B**) Supernatants were collected and the production of IL-12 and TNF-α was measured by ELISA. The concentrations of cytokines are shown. Data are from 3 independent experiments. *p* values are indicated (paired t-test).

Our previous study showed that *Pleurotus ferulae* water extract could promote DC maturation via TLR4 signaling pathway^28^. Here, we also investigated whether GUWE promotes DC maturation via TLR4 signaling pathway. DCs were pretreated with TLR4 inhibitor, TAK-242, and then treated with different concentrations of GUWE or LPS. After 12 h, DCs were collected to analyze the expressions of CD40 and CD86 by flow cytometry and the supernatant was collected to detect the production of IL-12 and TNF-α by ELISA. Upon TAK-242 pretreatment, the expressions of CD40 and CD86 (Fig. 3A), and the production of IL-12 and TNF-α (Fig. 3B) induced by LPS and GUWE were significantly inhibited, suggesting that GUWE promotes DC maturation and cytokine production via TLR4 signaling pathway.

**Figure 4:**
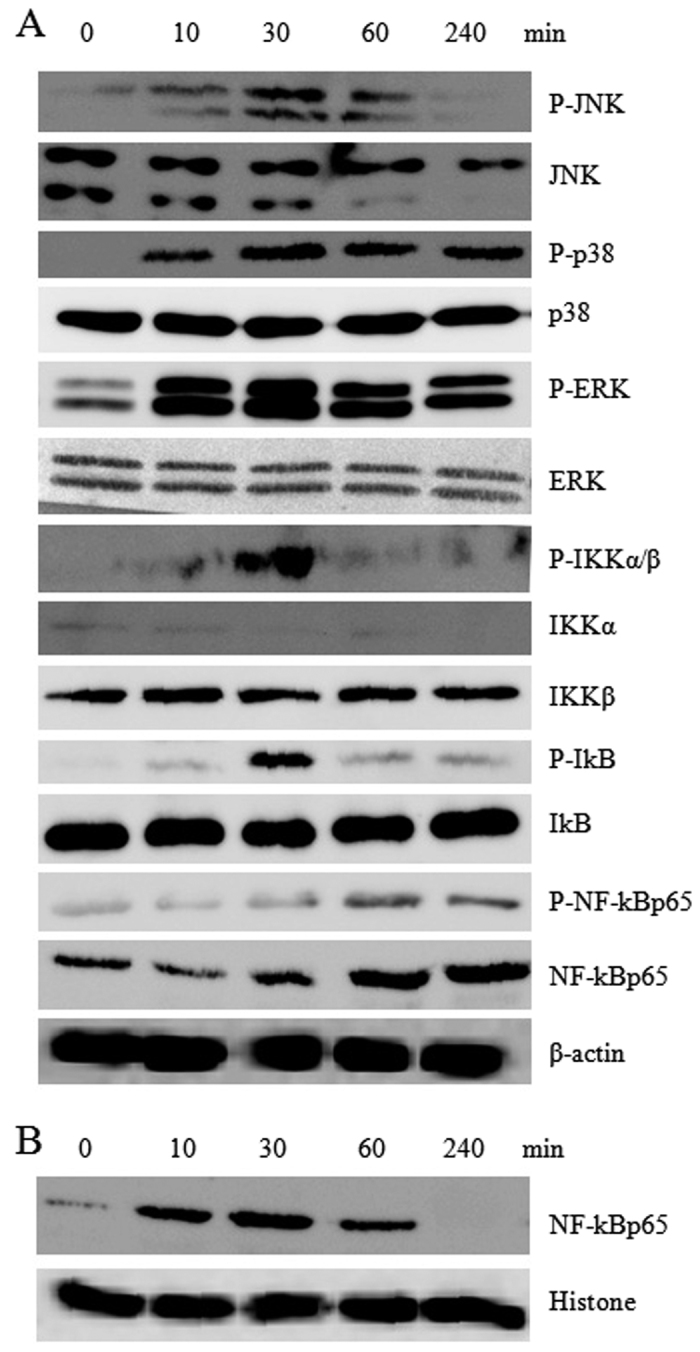
The effect of GUWE on MAPK and NF-κB signaling pathways in DCs. DCs were treated with 40 μg/ml of GUWE, then nuclear and cytoplasmic proteins were isolated at the indicated time points. The levels of protein and their phosphorylation in cytoplasm (**A**) or nuclei (**B**) were detected by Western blot. Cropped blots are shown and full-length blots are included in the Supplementary Information.

We further detected the down-stream molecules of MAPK and NF-κB signaling pathways. DCs were treated with 40 μg/ml of GUWE for different times (0, 10, 30, 60 and 240 min) and the cytoplasmic and nuclear proteins were extracted. The protein levels and their phosphorylation levels were detected by Western blot. We found that the phosphorylation levels of JNK, p38, ERK, IKKα/β, IκB and NF-κBp65 were increased in 10 min of GUWE treatment and arrived at the maximum at 30 min (Fig. 4A). Consistently, the levels of NF-κBp65 was increased in nuclei (Fig. 4B). The results suggested that GUWE activated MAPK and NF-κB signaling pathways through TLR4.

### HPV DC-based vaccines suppressed tumor growth in tumor mouse model

**Figure 5:**
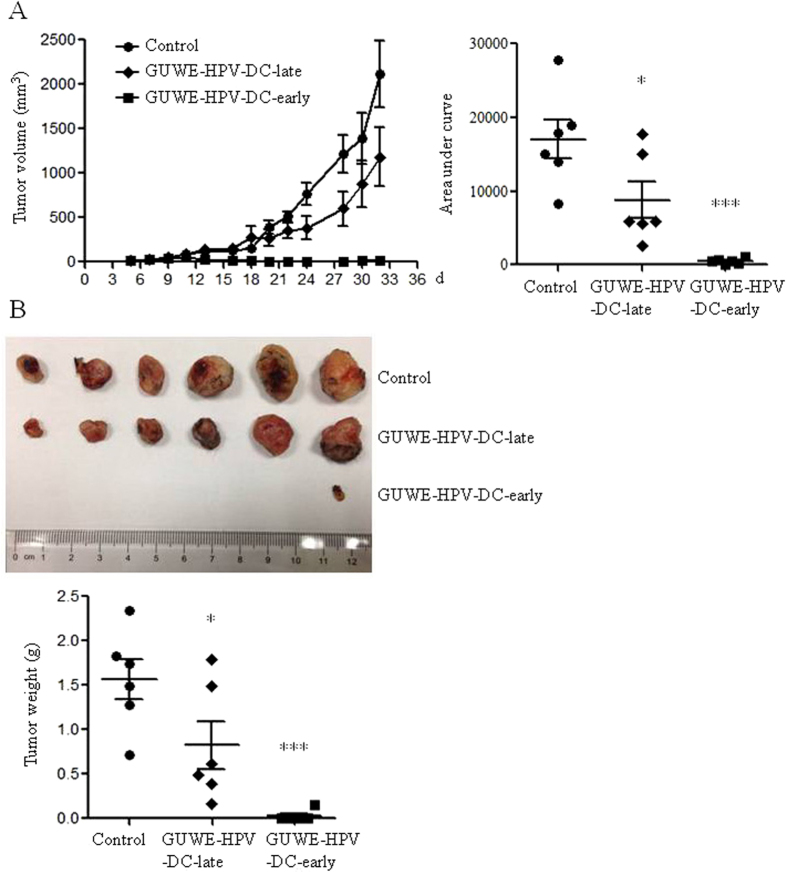
Tumor growth and weight after treatment with GUWE-HPV-DCs. Tumor mice were immunized with GUWE-HPV-DCs on day 5 or 12 after injection of TC-1 cells. (**A**) Tumor volumes were measured. The data are shown in the left panel. The area under curve was calculated with Prism 5 and the values are shown in right panel. (**B**) Tumors were isolated and weighted 32 days after tumor induction. The tumor photo and weight are shown in upper and lower panels, respectively. *p < 0.05 and ***p < 0.001 (ANOVA) compared to control group.

Our previous studies showed that PFWE could enhance the maturation and function of DCs, and the antitumor efficacy of HPV DC-based vaccine^28^,^29^. Here, we prepared the HPV DC-based vaccine as the following: GUWE treated DCs were pulsed with HPV-16 E6/E7 peptides (GUWE-HPV-DCs). The antitumor effect of GUWE-HPV-DCs was detected in TC-1 tumor mouse model. Tumor mice were treated with GUWE-HPV-DCs on day 5 or 12 after injection of TC-1 cells and named as GUWE-HPV-DC-early and GUWE-HPV-DC-late, respectively. Tumors were measured every other day from 5 days of TC-1 cell injection. Compared to control group, both experimental groups significantly inhibited tumor growth (Fig. 5A). Moreover, GUWE-HPV-DC-early group showed the best inhibitory effect on tumor growth and almost tumors could not be measured after 13 days. After 32 days, mice were sacrificed and tumors were isolated and weighted. Five out of 6 mice were tumor-free and only one mouse was found a small tumor-like tissue in GUWE-HPV-DC-early group. All of 6 mice were detected tumors in GUWE-HPV-DC-late group but the tumor weight was significantly reduced compared to control group (Fig. 5B).

### HPV DC-based vaccine generated antigen-specific cellular responses

**Figure 6:**
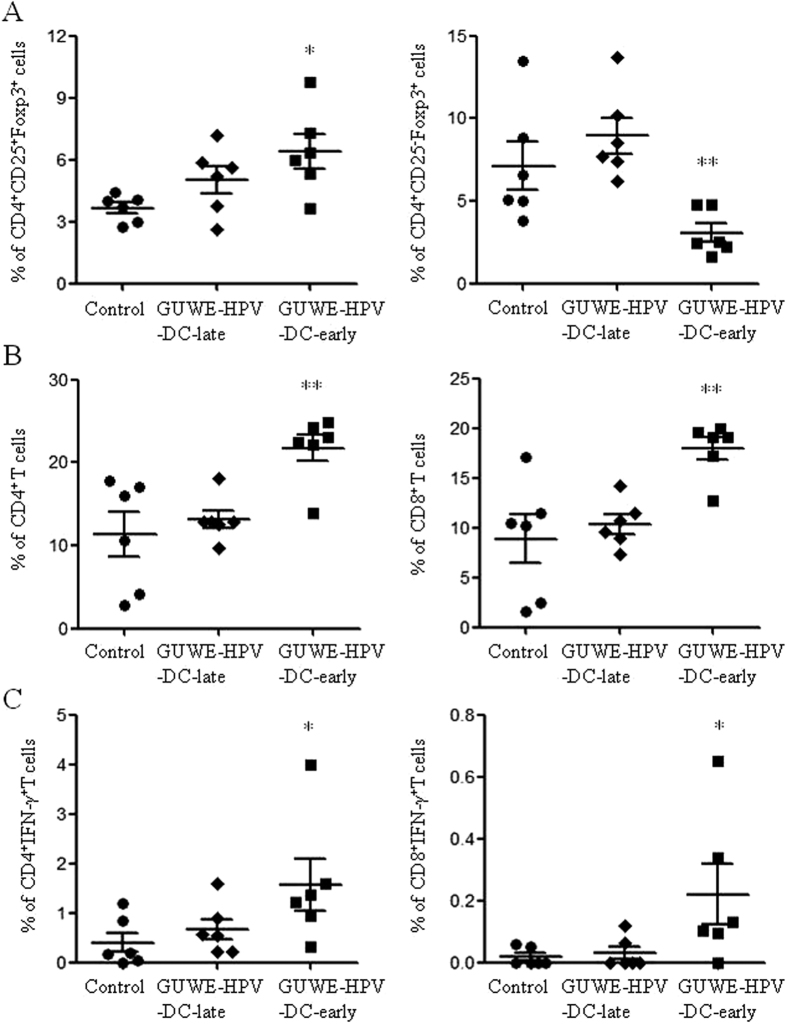
The frequencies of Tregs, CD4^+^ and CD8^+^ T cells, and CD4^+^IFN-γ^+^ and CD8^+^IFN-γ^+^ T cells after treatment with GUWE-HPV-DCs. Splenocytes were isolated 32 days after treatment with GUWE-HPV-DCs. The freshly isolated splenocytes were used to analyze the frequencies of Tregs (**A**) and CD4^+^ and CD8^+^ T cells (**B**). (**C**) Splenocytes were stimulated with HPV-16 E6/E7 peptides overnight. The frequency of antigen specific CD4^+^IFN-γ^+^ and CD8^+^IFN-γ^+^ T cells was analyzed by flow cytometry. *p < 0.05 and **p < 0.01 (ANOVA) compared to control group.

At the end of this experiment, freshly isolated splenocytes were used to analyze the frequencies of Tregs and CD4^+^ and CD8^+^ T cells. We found that the frequencies of natural Tregs (nTregs: CD4^+^CD25^+^Foxp3^+^) and induced Tregs (iTregs: CD4^+^CD25^−^Foxp3^+^) were significantly increased and decreased in GUWE-HPV-DC-early group compared to control group (Fig. 6A), respectively. But they have no significant difference between GUWE-HPV-DC-late and control groups. We also observed that the frequencies of CD4^+^ and CD8^+^ T cells were significantly increased in GUWE-HPV-DC-early group compared to control group (Fig. 6B). After HPV-16 E6/E7 peptides treatment, antigen-specific cellular responses were detected. The results showed that GUWE-HPV-DC-early induced HPV-specific CD4^+^ and CD8^+^ T cell responses (Fig. 6C). We also analyzed the correlation of HPV-specific CD8^+^ T cell responses among the frequencies of iTregs and tumor weight. The frequencies of HPV-specific CD8^+^ T cells are negatively correlated with the frequencies of iTregs and tumor weight (Supplementary Fig. S5). These results indicated that the reduction of iTregs might be contributed to the induction of HPV-specific cellular responses to inhibit tumor growth.

## Discussion

In this study, we found that GUWE promoted DC maturation and increased cytokine production through TLR4 signaling pathways. GUWE-HPV-DC-early induced HPV-specific cellular responses and suppressed tumor growth in TC-1 tumor mouse model.

Lots of efforts were focused on the improvement of the immune responses induced by DC-based vaccines through decreasing IL-10 production, increasing IL-12 production or improving DC survival^30^,^31^. Many studies including ours have shown that herbal medicine extracts or its components can enhance antitumor immune responses through promoting the activation status of DCs^28^,^29^,^32^,^33^. In this study, we observed that GUWE improved the maturation and cytokine production of DCs, especially IL-12 production. Consistently, GUWE-HPV-DC-early induced strong Th1 and CTL responses and totally suppressed tumor growth in TC-1 tumor mouse model. However, the late therapeutic strategy (GUWE-HPV-DC-late) only induced low level of HPV-specific cellular response and partially inhibited tumor growth. Tumor can suppress the induction of tumor-specific immune responses through the inhibition of DC maturation^34^. In GUWE-HPV-DC-late group, tumors might affect the maturation and function of *ex vivo*-matured DCs, which could not induce strong immune responses to inhibit tumor growth. In the future study, we will investigate the effect of GUWE and DC-based vaccine co-administration on the antitumor effect.

Tumors could induce Tregs to suppress antitumor immune responses^35^,^36^. We observed that the frequencies of iTregs in GUWE-HPV-DC-early group were significantly reduced compared to control group, which might contribute the induction of HPV-specific cellular responses. Similarly, our previous study found that the early therapeutic strategy reduced the frequencies of iTregs and induced strong HPV-specific cellular responses to inhibit the tumor growth^29^. We observed that the level of CD8^+^ T cell responses was negatively correlated with the frequencies of iTregs and the weight of tumor. One possible reason is that GUWE-HPV-DCs induce strong CD8^+^ T cell responses to inhibit tumor growth, which cannot promote the differentiation of iTregs. Another possible reason is that GUWE-HPV-DCs can inhibit the differentiation of iTregs to promote the generation of CD8^+^ T cell responses to inhibit tumor growth. However, the frequencies of nTregs in GUWE-HPV-DC-early group were increased, which is different with our previous study^29^. We further observed that the frequencies of both iTregs and nTregs in GUWE-HPV-DC-late group were similar with control group, which might be another reason for the weak HPV-specific cellular responses in GUWE-HPV-DC-late group. In our previous study, the late therapeutic strategy still reduced the frequencies of both iTregs and nTregs^29^. The different time points and doses of DC vaccination might be caused the difference in the frequencies of Tregs between the two studies.

HPV DC-based vaccines are good candidates for the therapy of HPV infection caused cervical cancers, due to which could induce antigen-specific cellular immune responses and showed the clinical benefit^37^,^38^. However, the clinical efficacy needs to be improved. Disease stage is strongly correlated with the survival of cancer patients^39^. Due to the lack of the screening programs, cervical cancers were usually diagnosed at high disease stages in developing countries. Therefore, we carried out both the early and late therapies and found that both therapies could significantly inhibit tumor growth. However, the late therapy only partially suppressed tumor growth, which might be due to the weak cellular immune responses and the suppressive microenvironment induced by tumors. In the future study, we will increase the doses of DCs vaccination to enhance the immune responses and antitumor efficacy. The combined strategies present the future direction for tumor therapy. Therefore, the combination of GUWE-HPV-DCs with other strategies including radiotherapy, chemotherapy, adjuvants and inhibitors of Tregs or immune check-point deserved to be explored in the future.

In conclusion, GUWE promotes the maturation and cytokine production of DCs via TLR4 and the down-stream MAPK and NF-κB signaling pathways. GUWE-HPV-DCs induced HPV-specific cellular responses and suppressed tumor growth in TC-1 tumor mouse model, especially for the early therapy, suggesting that GUWE-HPV-DCs might be a good strategy to treat cervical cancer caused by HPV infection.

## Materials and Methods

### The preparation of *G. uralensis* water extract (GUWE) and endotoxin detection

The root of *G. uralensis* Fisch was collected from Yili in Xinjiang province, China. 50 g of dry minced root was extracted three times with 500 ml of distilled water with stirring at 60 °C for 2 h. The extracts were pooled together and filtered through Whatman no. 4 filter paper. The supernatant was concentrated using a rotary vacuum evaporator at 40 °C, and precipitated with 4 volumes of ethanol at 4 °C overnight. After spinning down at 8000 rpm for 15 min, the pellet was collected and the remaining solvent was removed with a freeze-drier. The dry powder of GUWE was constituted in distilled water and sterilized with a 0.22 μm filter. The content of polysaccharides was measured by anthrone-sulphuric acid method.

The endotoxin in GUWE was detected by Gel Clot TAL assay (Catalog number G010250) according to the manufacturer’s instruction (Xiamen BioEndo Technology, Co., Ltd). 2λ endotoxin standard was used as positive control. Endotoxin-free water was used as negative control. The detection sensitivity is 0.25 EU/ml.

### The qualitative analysis of GUWE by LC (HPLC)-MS (Mass Spectrometer)/MS system

LC-MS/MS was carried out by Key Laboratory of Xinjiang Indigenous Medicinal Plants Resource Utilization, Xinjiang Technical Institute of Physics and Chemistry, Chinese Academy of Sciences according to their previous protocol^40^. The standard glycyrrhizin (glycyrrhizic acid) was purchased from ChemCatch and its purity was ≥99%. The solutions of glycyrrhizin and GUWE were separated by Agilent 1200 series HPLC system (Agilent Technologies, Waldbron, Germany) equipped with a Phenomenon C18 column (5 μm, 4.6 × 250 mm) at column temperature of 35 °C. The mobile phase was composed of A (acetonitrile) and B (0.1% formic acid). 10 μl of each sample in methanol was injected by an auto-sampler and eluted at a flow rate of 1 ml/min by the following gradient of mobile phase (time in minutes; % of A): (0; 40), (10; 40), (15; 100), (25; 100).

Mass spectrometry was conducted using a QSTAR Elite LC/MS/MS system (Applied Biosystems/MDS Sciex, Canada) equipped with an electrospray ionization (ESI) ion source according to previous description^40^. The negative ion mode was used under the optimized parameters: ESI voltage at −4500 V, nebulizer gas at 60 psi, auxiliary gas at 50 psi, curtain gas at 35 psi, Turbo gas temperature at 450 °C, declustering potential at −60 V, focusing potential at −350 V, declustering potential 2 at −10 V. IDA (Information-Dependent Acquisition) method was used to analyze the samples, which can automatically select candidate ions for MS/MS assay. The TOF (time-of-flight) mass range was set from m/z 100 to 1500. The mass analyzer was calibrated using taurocholic acid (2 ng/μl) with a syringe pump at a flow rate of 5 μl/min. Analyst QS 2.0 software were used to acquire and process data.

### Animals

BALB/c and C57BL/6 female mice (6–8 weeks) were obtained from the Beijing laboratory animal research center (Beijing, China). All mice were housed in a temperature-controlled and light-cycled animal facility of Xinjiang University.

### Ethics statement

All methods were carried out in accordance with the guidelines approved by Xinjiang University. All animal experiments were approved by the Committee on the Ethics of Animal Experiments of Xinjiang Key Laboratory of Biological Resources and Genetic Engineering and performed under the guidelines of the Animal Care and Use Committee of College of Life Science and Technology, Xinjiang University.

### The induction of bone marrow-derived DCs

Immature DCs were induced from bone marrow of mice by Granulocyte/Macrophage Colony-Stimulating Factor (GM-CSF) according to previous description^28^. Briefly, bone marrow cells were collected from C57BL/6 mice and cultured in RPMI-1640 contained 10% heat-inactivated fetal bovine serum (FBS), 2 mM L-glutamine, 100 units/ml penicillin-streptomycin, 50 μM β-mercaptoethanol and 20 ng/ml GM-CSF. On day 7, non-adherent cells (1 × 10^6^/ml) were collected and treated with 0, 4, 20 and 40 μg/ml of GUWE, or 20 ng/ml of LPS (Sigma-Aldrich) for 12 h. For endocytosis experiment, GUWE and LPS treated DCs were inoculated with FITC-Dextran (Sigma-Aldrich) for 1 h and analyzed by flow cytometry. For TLR4 inhibitor experiments, DCs were pretreated with 1 μM TAK-242 (Medchem-express) for 1 h, and then treated with 0, 4, 20 and 40 μg/ml of GUWE or 20 ng/ml of LPS for 12 h.

### Detection of cytokine production by enzyme-linked immunosorbent assay (ELISA)

The supernatant was collected from the above cultured DCs with different treatments and the cytokine production (IL-1β, IL-6, IL-12 and TNF-α) was detected by ELISA using ELISA kit according to the manufacturer’s instruction (Elabscience, China). Absorbance at 450 nm was measured using an ELISA plate reader (Bio-Rad, USA).

### Mixed lymphocyte reaction (MLR)

On day 7, DCs from C57BL/6 mice were treated with 0, 4, 20 and 40 μg/ml of GUWE or 20 ng/ml of LPS for 12 h, and then treated with 10 μg/ml of mitomycin C for 1 h. These DCs were co-cultured with splenocytes from BALB/c mice at the ratios of 1:10 and 1:5 in a 37 °C incubator with a 5% CO_2_ atmosphere. After 48 h, cell proliferation was analyzed by 3-(4, 5-dimethylthiazol-2-yl)-2, 5-diphenyltetrazolium bromide (MTT) (Sigma-Aldrich) assay. The supernatant was discarded after centrifugation at 1200 rpm for 5 min and then 100 μl of MTT solution (1 mg/ml in RPMI-1640 medium) was added to each well. After 4 h, 100 μl DMSO was added to dissolve the formed formazan crystals. The OD_490_ values were measured by a 96-well microplate reader (Bio-Rad Laboratories, CA, USA).

### Western blot

The antibodies against IKKα, IKKβ, IkB, JNK, p38, ERK, NF-kBp65 and their phosphorylated antibodies were purchased from Cell Signaling Technology. Anti-β-actin and anti-histone were purchased from Beijing ComWin Biotech Co., Ltd (Beijing, China). Anti-mouse IgG-HRP and anti-rabbit IgG-HRP were obtained from Cell Signaling Technology.

DCs were treated with 40 μg/ml of GUWE for 0, 10, 30, 60 and 240 min and proteins were extracted using Nuclear and Cytoplasmic Protein Extraction Kit (Beijing ComWin Biotech Co., Ltd), then the protein concentration was determined by BCA Kit (Thermo Fisher Scientific, USA) according to the manufacturer’s instructions. Equal amount of proteins for each sample were transferred to PVDF membranes after isolation by 12% SDS-PAGE. After blocking with TBST buffer (20 mmol/L Tris-HCl, 150 mmol/L NaCl, 0.05% Tween 20) contained 5% nonfat milk for 1 h at RT, primary antibodies were added and incubated on shaker overnight at 4 °C, followed the incubation with horseradish peroxidase-conjugated secondary antibodies for 1 h at RT. After extensive washing with TBST, the target proteins were detected using ECL assay kit (Beyotime Biotechnology Co., Ltd, China).

### Preparation of HPV DC vaccine

HPV DC vaccine was prepared according to our previous description^29^. Briefly, DCs were treated with 40 μg/ml of GUWE for 12 h, and washed with medium, then pulsed with 10 μg/ml of HPV-16 E6/E7 peptides including E6_43–57_ (QLLRREVYDFAFRDL), E6_53–62_ (AFRDLCIVYR), E7_11–20_ (YMLDLQPETT), E7_44–62_ (QAEPDRAHYNIVTFCCKCD) and E7_81–94_ (DLLMGTLGIVCPIC) for 2 h. After washing with PBS, DCs were re-suspended in PBS at the concentration of 1 × 10^7^/ml, which were named as HPV-DCs.

### TC-1 Tumor model and treatment

TC-1 cells, which expressed HPV-16 E6 and E7^41^, in log-phase growth were collected and washed with PBS. 1 × 10^6^/ml of TC-1 cells were re-suspended in PBS and 100 μl of TC-1 cells were subcutaneously injected into the right flank of C57BL/6 mice. Tumor mice were randomly divided into three groups (6 mice/group) and intradermally injected with 50 μl PBS (control) or immunized with 5 × 10^5^ HPV-DCs in 50 μl PBS on day 5 (early therapy) or 12 (late therapy). Tumors were measured every other day using calipers and tumor volumes were calculated using the formula: tumor volume (mm^3^) = (length × width^2^)/2. At the end of tumor study, tumors were isolated and weighted. Splenocytes were used to detect the immune responses by flow cytometry.

### Flow cytometry

The viability of DCs was analyzed by Annexin V/PI staining kit (Shanghai Yeasen Biotechnology Co., Ltd., China) according to the manufacturer’s instruction. For evaluation of DC maturation, cell surface staining was performed using the pools of mAbs (BD Biosciences): PE-CD11c, APC-CD40 and FITC-CD80 or APC-CD11c, FITC- CD86 and PE-MHC II. The frequencies of regulatory T cells (Tregs), CD4^+^ and CD8^+^ T cells in spleens of tumor mice were analyzed according to our previous description^29^. For analysis of cellular responses, splenocytes (1 × 10^6^/ml) were treated with HPV-16 E6/E7 peptides and cultured overnight in the presence of Golgi stop (BD Biosciences). Cell surface and intracellular staining was performed according to our previous description^29^. All samples were collected on FACSCalibur (BD Biosciences) and the data were analyzed by the FlowJo platform (Tree Star, Inc., Ashland, OR).

### Statistical analysis

Statistical significance was analyzed by one-way analysis of variance (ANOVA) or paired t-test. A value of *p* < 0.05 was considered to be statistically significant.

## Additional Information

**How to cite this article:** Aipire, A. *et al. Glycyrrhiza uralensis* water extract enhances dendritic cell maturation and antitumor efficacy of HPV dendritic cell-based vaccine. *Sci. Rep.* **7**, 43796; doi: 10.1038/srep43796 (2017).

**Publisher's note:** Springer Nature remains neutral with regard to jurisdictional claims in published maps and institutional affiliations.

## Supplementary Material

Supplementary Figures

## References

[b1] Bosch F. X., Lorincz A., Munoz N., Meijer C. J. & Shah K. V. The causal relation between human papillomavirus and cervical cancer. Journal of clinical pathology 55, 244–265 (2002). 11919208 10.1136/jcp.55.4.244 PMC1769629

[b2] Bosch F. X. . Prevalence of human papillomavirus in cervical cancer: a worldwide perspective. International biological study on cervical cancer (IBSCC) Study Group. Journal of the National Cancer Institute 87, 796–802 (1995). 7791229 10.1093/jnci/87.11.796

[b3] Yang A. . Current state in the development of candidate therapeutic HPV vaccines. Expert review of vaccines, 1–19, doi: 10.1586/14760584.2016.1157477 (2016). PMC4977850 26901118

[b4] Bosch F. X. . Comprehensive control of human papillomavirus infections and related diseases. Vaccine 31 Suppl 7, H1–31, doi: 10.1016/j.vaccine.2013.10.003 (2013). 24332295 PMC7605442

[b5] Villa L. L. . Prophylactic quadrivalent human papillomavirus (types 6, 11, 16, and 18) L1 virus-like particle vaccine in young women: a randomised double-blind placebo-controlled multicentre phase II efficacy trial. The Lancet. Oncology 6, 271–278, doi: 10.1016/S1470-2045(05)70101-7 (2005). 15863374

[b6] Paavonen J. . Efficacy of a prophylactic adjuvanted bivalent L1 virus-like-particle vaccine against infection with human papillomavirus types 16 and 18 in young women: an interim analysis of a phase III double-blind, randomised controlled trial. Lancet 369, 2161–2170, doi: 10.1016/S0140-6736(07)60946-5 (2007). 17602732

[b7] Forman D. . Global burden of human papillomavirus and related diseases. Vaccine 30 Suppl 5, F12–23, doi: 10.1016/j.vaccine.2012.07.055 (2012). 23199955

[b8] Arbyn M. . Worldwide burden of cervical cancer in 2008. Annals of oncology: official journal of the European Society for Medical Oncology/ESMO 22, 2675–2686, doi: 10.1093/annonc/mdr015 (2011). 21471563

[b9] Lee S. J., Yang A., Wu T. C. & Hung C. F. Immunotherapy for human papillomavirus-associated disease and cervical cancer: review of clinical and translational research. Journal of gynecologic oncology 27, e51, doi: 10.3802/jgo.2016.27.e51 (2016). 27329199 PMC4944018

[b10] Kalinski P. Dendritic cells in immunotherapy of established cancer: Roles of signals 1, 2, 3 and 4. Curr Opin Investig Drugs 10, 526–535 (2009). PMC2919813 19513941

[b11] Orsini E., Guarini A., Chiaretti S., Mauro F. R. & Foa R. The circulating dendritic cell compartment in patients with chronic lymphocytic leukemia is severely defective and unable to stimulate an effective T-cell response. Cancer Res 63, 4497–4506 (2003). 12907623

[b12] Della Bella S. . Altered maturation of peripheral blood dendritic cells in patients with breast cancer. British journal of cancer 89, 1463–1472, doi: 10.1038/sj.bjc.6601243 (2003). 14562018 PMC2394334

[b13] Constantino J., Gomes C., Falcao A., Cruz M. T. & Neves B. M. Antitumor dendritic cell-based vaccines: lessons from 20 years of clinical trials and future perspectives. Translational research: the journal of laboratory and clinical medicine 168, 74–95, doi: 10.1016/j.trsl.2015.07.008 (2016). 26297944

[b14] Sabado R. L. & Bhardwaj N. Dendritic cell immunotherapy. Ann N Y Acad Sci 1284, 31–45, doi: 10.1111/nyas.12125 (2013). 23651191

[b15] Verdijk P., Aarntzen E. H., Punt C. J., de Vries I. J. & Figdor C. G. Maximizing dendritic cell migration in cancer immunotherapy. Expert Opin Biol Ther 8, 865–874, doi: 10.1517/14712598.8.7.865 (2008). 18549318

[b16] Steinman R. M. & Banchereau J. Taking dendritic cells into medicine. Nature 449, 419–426, doi: 10.1038/nature06175 (2007). 17898760

[b17] Rolinski J. & Hus I. Breaking immunotolerance of tumors: A new perspective for dendritic cell therapy. J Immunotoxicol, doi: 10.3109/1547691X.2013.865094 (2014). 24495309

[b18] Macatonia S. E. . Dendritic cells produce IL-12 and direct the development of Th1 cells from naive CD4+ T cells. J Immunol 154, 5071–5079 (1995). 7730613

[b19] Carreno B. M. . IL-12p70-producing patient DC vaccine elicits Tc1-polarized immunity. J Clin Invest 123, 3383–3394, doi: 10.1172/JCI68395 (2013). 23867552 PMC3726168

[b20] Vacchelli E. . Trial watch: Dendritic cell-based interventions for cancer therapy. Oncoimmunology 2, e25771, doi: 10.4161/onci.25771 (2013). 24286020 PMC3841205

[b21] Schreibelt G. . Commonly used prophylactic vaccines as an alternative for synthetically produced TLR ligands to mature monocyte-derived dendritic cells. Blood 116, 564–574, doi: 10.1182/blood-2009-11-251884 (2010). 20424184

[b22] Chen X., Yang L., Howard O. M. & Oppenheim J. J. Dendritic cells as a pharmacological target of traditional Chinese medicine. Cell Mol Immunol 3, 401–410 (2006). 17257493

[b23] Li J., Li J. & Zhang F. The immunoregulatory effects of Chinese herbal medicine on the maturation and function of dendritic cells. Journal of ethnopharmacology 171, 184–195, doi: 10.1016/j.jep.2015.05.050 (2015). 26068430

[b24] Asl M. N. & Hosseinzadeh H. Review of pharmacological effects of Glycyrrhiza sp. and its bioactive compounds. Phytotherapy research: PTR 22, 709–724, doi: 10.1002/ptr.2362 (2008). 18446848 PMC7167813

[b25] Tang Z. H. . A Systematic Review of the Anticancer Properties of Compounds Isolated from Licorice (Gancao). Planta medica 81, 1670–1687, doi: 10.1055/s-0035-1558227 (2015). 26695708

[b26] Cho H. J. . Hexane/ethanol extract of Glycyrrhiza uralensis licorice exerts potent anti-inflammatory effects in murine macrophages and in mouse skin. Food chemistry 121, 959–966 (2010).

[b27] Fu Y., Chen J., Li Y., Zheng Y. & Li P. Antioxidant and anti-inflammatory activities of six flavonoids separated from licorice. Food chemistry 141, 1063–1071 (2013). 23790887 10.1016/j.foodchem.2013.03.089

[b28] Li J. . Pleurotus ferulae water extract enhances the maturation and function of murine bone marrow-derived dendritic cells through TLR4 signaling pathway. Vaccine 33, 1923–1933, doi: 10.1016/j.vaccine.2015.02.063 (2015). 25748337

[b29] Li J. . The combination of Pleurotus ferulae water extract and CpG-ODN enhances the immune responses and antitumor efficacy of HPV peptides pulsed dendritic cell-based vaccine. Vaccine, doi: 10.1016/j.vaccine.2016.05.022 (2016). 27211038

[b30] Kim J. H. . Blocking the immunosuppressive axis with small interfering RNA targeting interleukin (IL)-10 receptor enhances dendritic cell-based vaccine potency. Clinical and experimental immunology 165, 180–189, doi: 10.1111/j.1365-2249.2011.04410.x (2011). 21592111 PMC3142643

[b31] Wang Y. T., Li W., Liu Q., Guan X. & Hu J. Dendritic cells treated with HPV16mE7 in a three-dimensional model promote the secretion of IL-12p70 and IFN-gamma. Experimental and molecular pathology 91, 325–330, doi: 10.1016/j.yexmp.2011.03.005 (2011). 21463625

[b32] Chang W. T. . Specific Dioscorea Phytoextracts Enhance Potency of TCL-Loaded DC-Based Cancer Vaccines. Evid Based Complement Alternat Med 2013, 932040, doi: 10.1155/2013/932040 (2013). 23935688 PMC3723319

[b33] Kim J. Y. . Adjuvant effect of a natural TLR4 ligand on dendritic cell-based cancer immunotherapy. Cancer letters 313, 226–234, doi: 10.1016/j.canlet.2011.09.009 (2011). 21974804

[b34] Ma Y., Shurin G. V., Peiyuan Z. & Shurin M. R. Dendritic cells in the cancer microenvironment. Journal of Cancer 4, 36–44, doi: 10.7150/jca.5046 (2013). 23386903 PMC3564245

[b35] Jaafar F. . Correlation of CXCL12 expression and FoxP3+ cell infiltration with human papillomavirus infection and clinicopathological progression of cervical cancer. The American journal of pathology 175, 1525–1535, doi: 10.2353/ajpath.2009.090295 (2009). 19808652 PMC2751549

[b36] Kryczek I. . FOXP3 defines regulatory T cells in human tumor and autoimmune disease. Cancer Res 69, 3995–4000, doi: 10.1158/0008-5472.CAN-08-3804 (2009). 19383912

[b37] Murakami M., Gurski K. J., Marincola F. M., Ackland J. & Steller M. A. Induction of specific CD8+ T-lymphocyte responses using a human papillomavirus-16 E6/E7 fusion protein and autologous dendritic cells. Cancer Res 59, 1184–1187 (1999). 10096544

[b38] Santin A. D., Bellone S., Gokden M., Cannon M. J. & Parham G. P. Vaccination with HPV-18 E7-pulsed dendritic cells in a patient with metastatic cervical cancer. N Engl J Med 346, 1752–1753, doi: 10.1056/NEJM200205303462219 (2002). 12037163

[b39] Quinn M. A. . Carcinoma of the cervix uteri. FIGO 26th Annual Report on the Results of Treatment in Gynecological Cancer. International journal of gynaecology and obstetrics: the official organ of the International Federation of Gynaecology and Obstetrics 95 Suppl 1, S43–103, doi: 10.1016/S0020-7292(06)60030-1 (2006). 17161167

[b40] Gu D., Yang Y., Abdulla R. & Aisa H. A. Characterization and identification of chemical compositions in the extract of Artemisia rupestris L. by liquid chromatography coupled to quadrupole time-of-flight tandem mass spectrometry. Rapid Commun Mass Spectrom 26, 83–100 (2012). 22215581 10.1002/rcm.5289

[b41] Lin K. Y. . Treatment of established tumors with a novel vaccine that enhances major histocompatibility class II presentation of tumor antigen. Cancer Res 56, 21–26 (1996). 8548765

