# Supplementary material for: Glycyrrhiza uralensis water extract enhances dendritic cell maturation and antitumor efficacy of HPV dendritic cell-based vaccine
Source: Sci Rep. 2017 Mar 8;7:43796. doi: 10.1038/srep43796 (PMC5341557; doi:10.1038/srep43796)
Supplement: Supplementary Figures [file srep43796-s1.pdf]

Glycyrrhiza uralensis water extract enhances dendritic cell maturation and antitumor efficacy of HPV dendritic cell-based vaccine

Adila Aipire[a], Jinyu Li[a], Pengfei Yuan[a], Jiang He[b], Yelang Hu[a], Lu Liu[c], Xiaoli Feng[c], Yijie Li[a], Fuchun Zhang[a], Jianhua Yang[a,d], Jinyao Li[a,]*

[a] Xinjiang Key Laboratory of Biological Resources and Genetic Engineering, College of Life Science and Technology, Xinjiang University, Urumqi 830046, China

[b] Key laboratory of Xinjiang Uighur Medicine, Xinjiang Institute of Materia Medica, 140 Xinhua South Road, Urumqi 830004, China

[c] XinJiang DingJu Biotech CO., LTD, 181 Xicai Road, Urumqi 830000, China

[d] Texas Children's Cancer Center, Department of Pediatrics, Dan L. Duncan Cancer Center, Baylor College of Medicine, TX 77030, USA

*Corresponding Author: College of Life Science and Technology, Xinjiang University, 666 Shengli Road, Urumqi 830046, China. Tel: +86-991-8583259; Fax: +86-991-8583517; E-mail: ljyxju@xju.edu.cn.

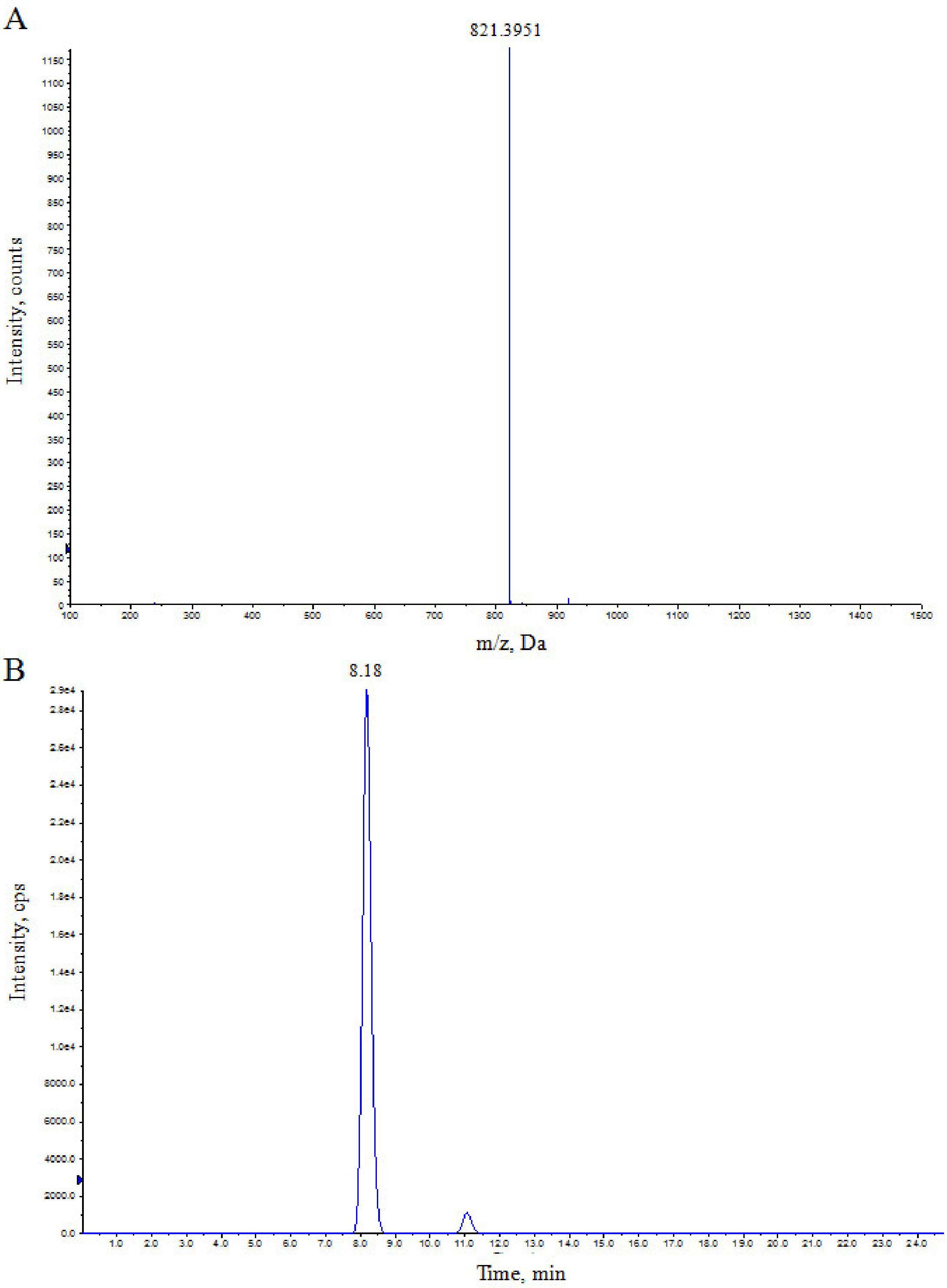
A
821.3951
Intensity, counts
m/z, Da
B
8.18
Intensity, cps
Time, min

C

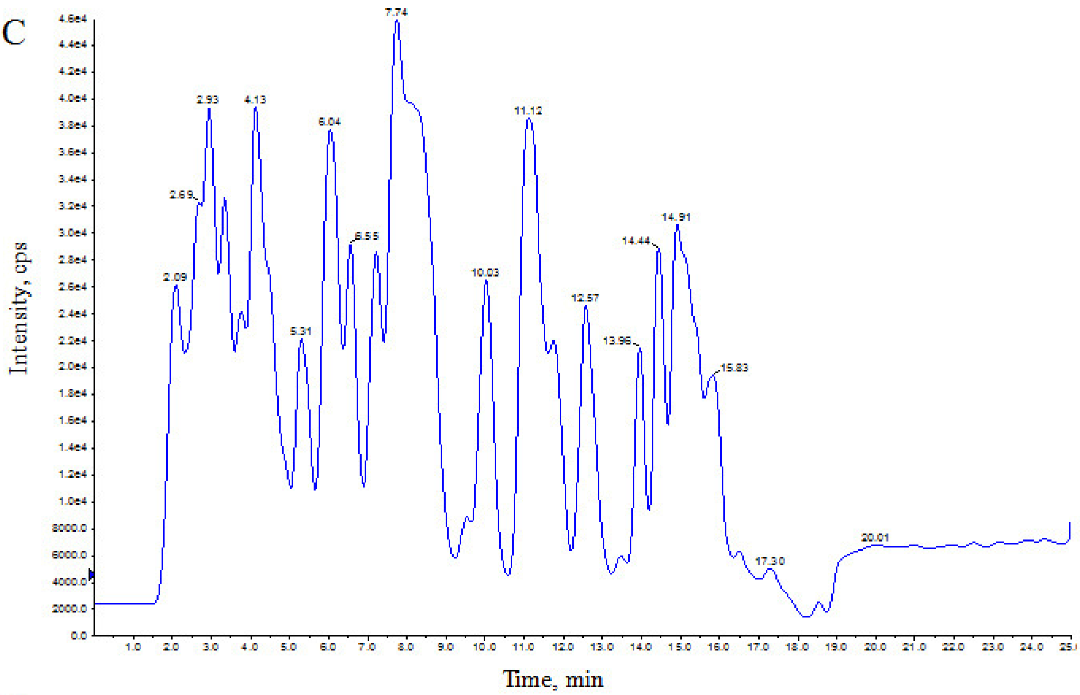

Intensity, cps
Time, min
7.74
2.93
4.13
6.04
11.12
2.69
14.91
14.44
6.55
2.09
10.03
12.57
5.31
13.96
15.83
17.30
20.01

D

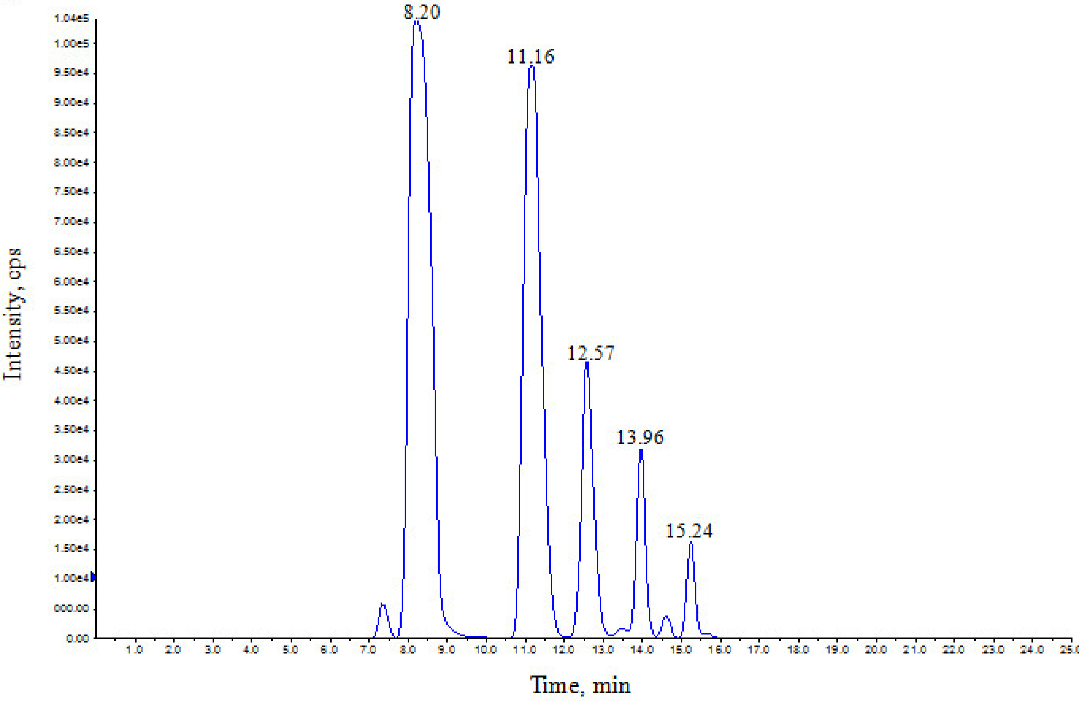

Intensity, cps
Time, min
8.20
11.16
12.57
13.96
15.24

E

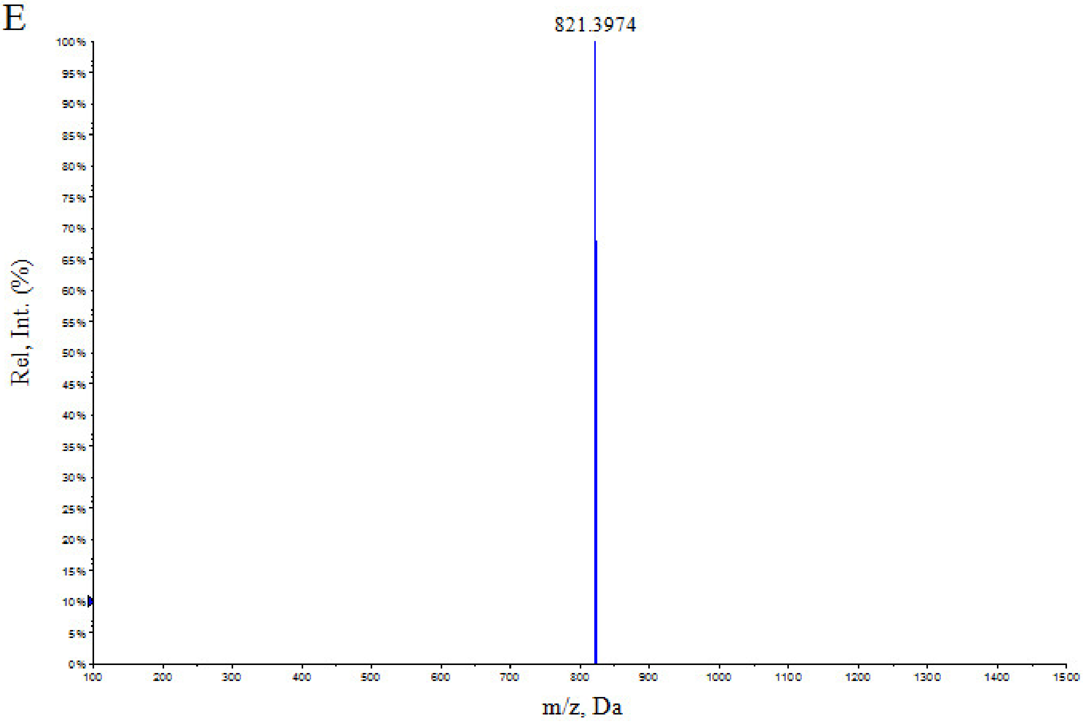

Supplementary Figure S1. The quality control of GUWE by LC-MS/MS. The standard glycyrrhizin and GUWE were analyzed by LC-MS/MS. (A) The m/z of glycyrrhizin is shown. (B) The retention time (t_R) of glycyrrhizin is shown. (C) The total ion chromatogram of GUWE is shown. (D) The retention times of components with m/z 821.4 in GUWE are shown. (E) The m/z of one component (t_R=8.2 min) is shown.

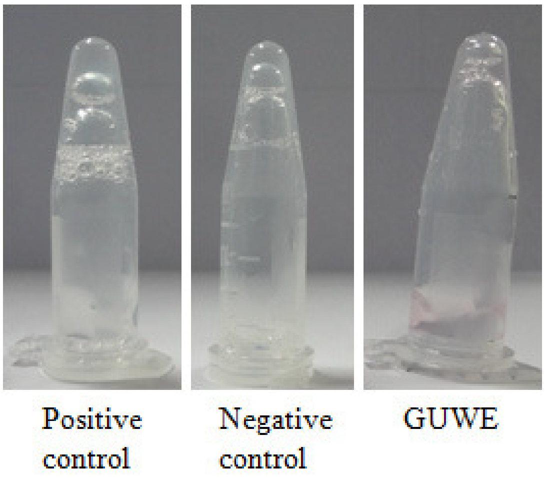

Supplementary Figure S2. Endotoxin detection of GUWE.

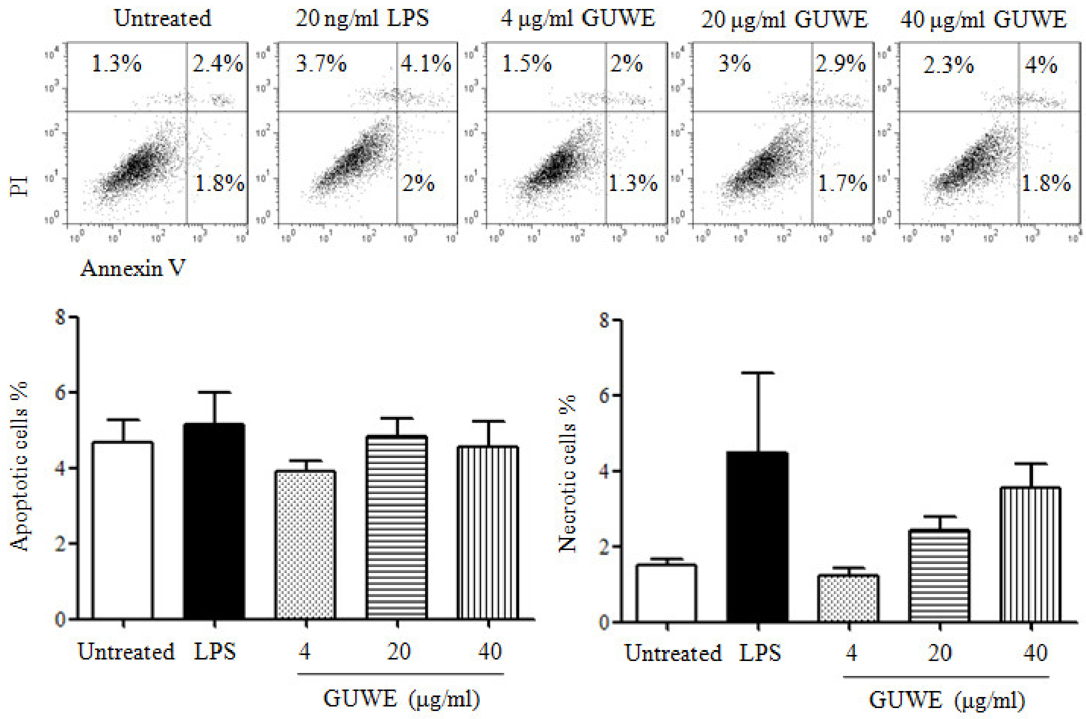

Supplementary Figure S3. The effect of GUWE on the viability of DCs. DCs were induced from bone marrow in the presence of GM-CSF. On day 7, DCs were treated with different concentrations of GUWE or LPS. After 12 h, DCs were collected and stained with Annexin V and PI. Samples were analyzed with flow cytometry. Representative dot plots are shown in upper panels. The frequencies of apoptosis and necrosis are shown in lower panels.

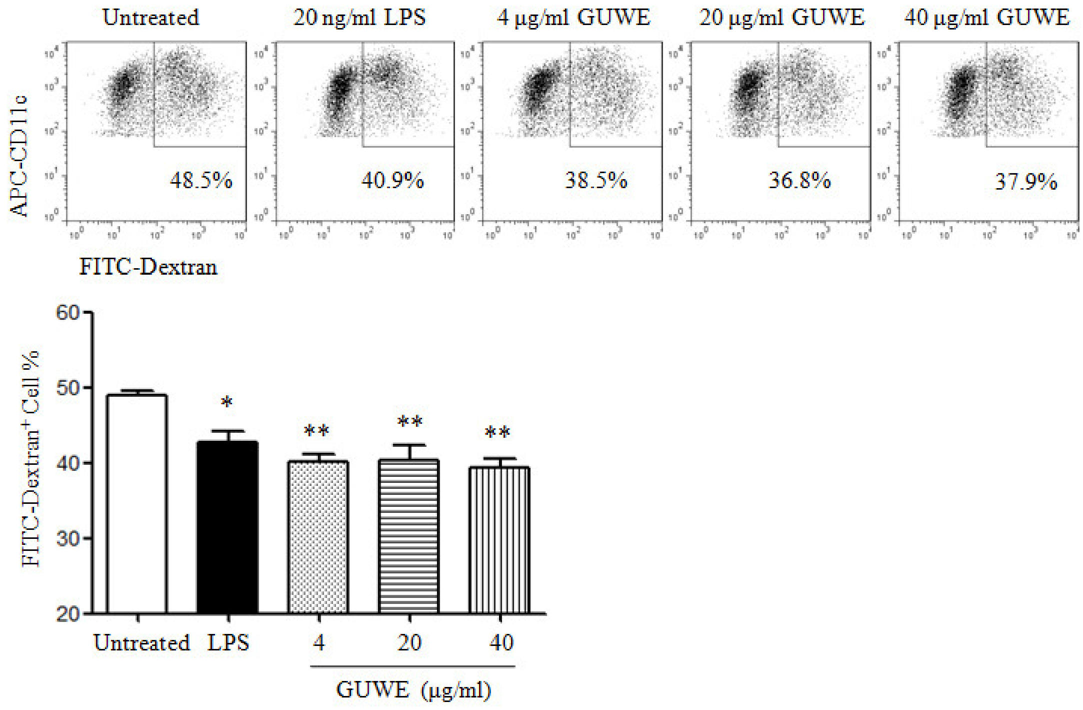

Supplementary Figure S4. The capacity of antigen up-take of DCs upon GUWE treatment. DCs were treated with different concentrations (4, 20 and 40 μg/ml) of GUWE or LPS for 12 h, and then inoculated with FITC-Dextran for 1 h. After staining with APC-CD11c, samples were analyzed with flow cytometry. The representative dot plots are shown in upper panels and the summary data (mean ± SEM) are shown in lower panel. Data are from 3 independent experiments and analyzed by ANOVA. * $p < 0.05$; ** $p < 0.01$ compared to untreated DCs.

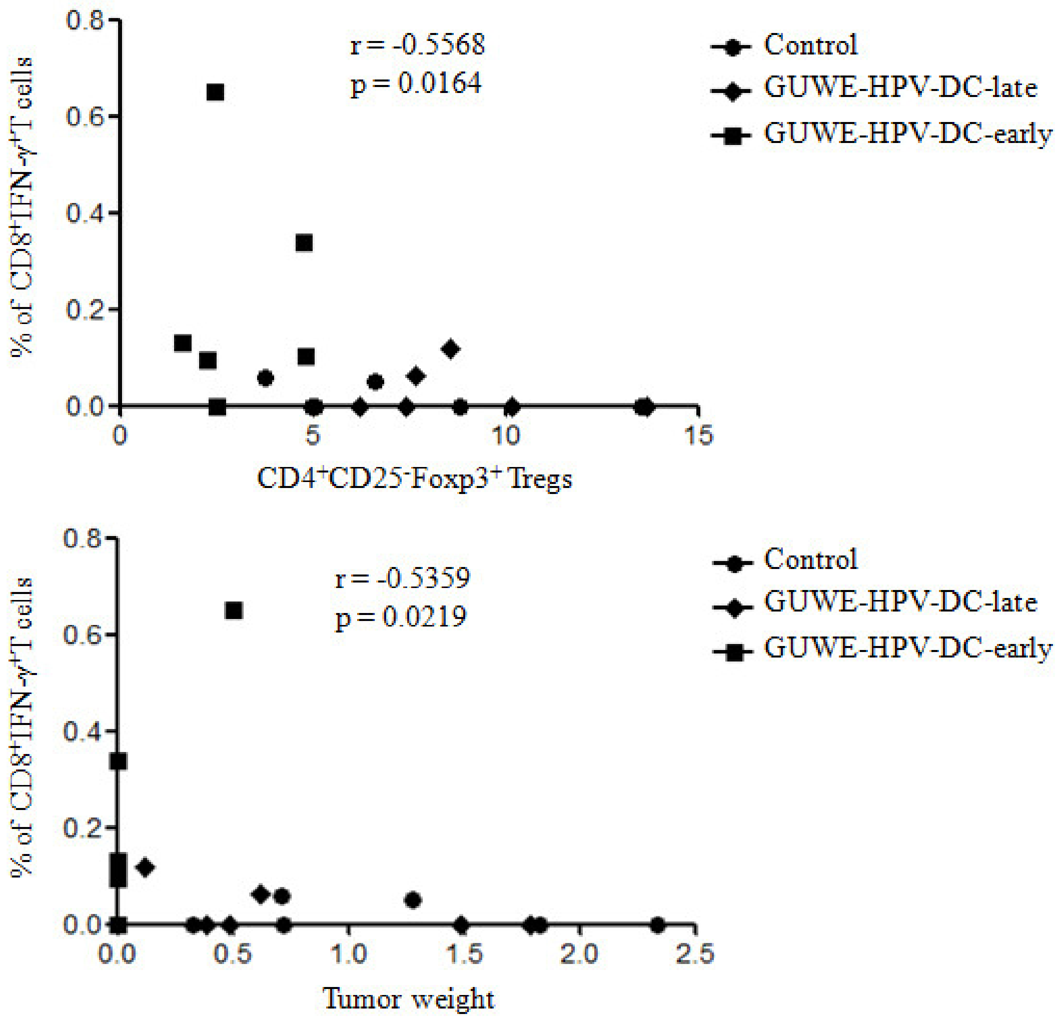

Supplementary Figure S5. The correlation of CD8$^+$IFN-γ$^+$ T cells with iTregs and tumor weight. The upper panel shows the correlation between CD8$^+$IFN-γ$^+$ T cells and iTregs. The lower panel shows the correlation between CD8$^+$IFN-γ$^+$ T cells and tumor weight.

Full-length blots for Figure 4.

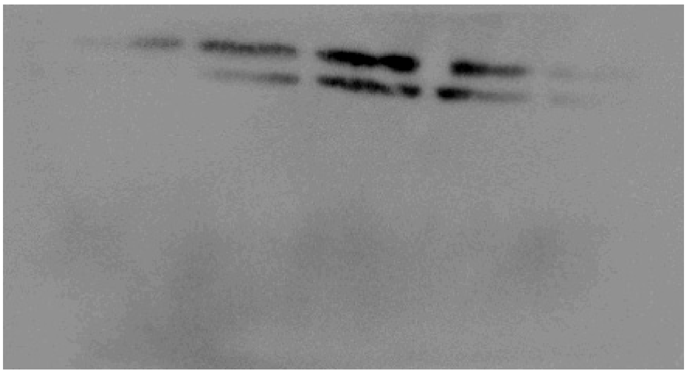

P-JNK

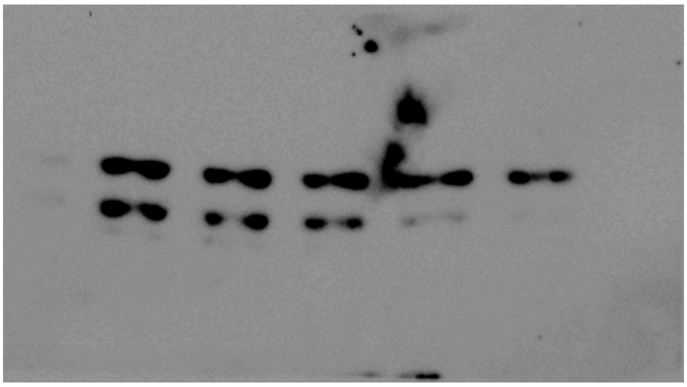

JNK

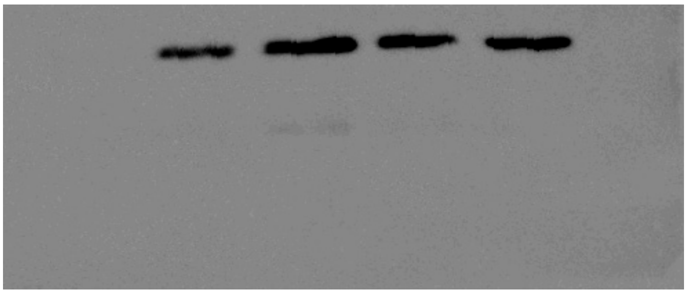

P-p38

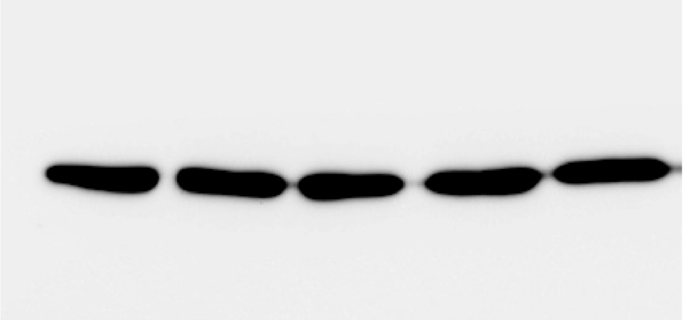

p38

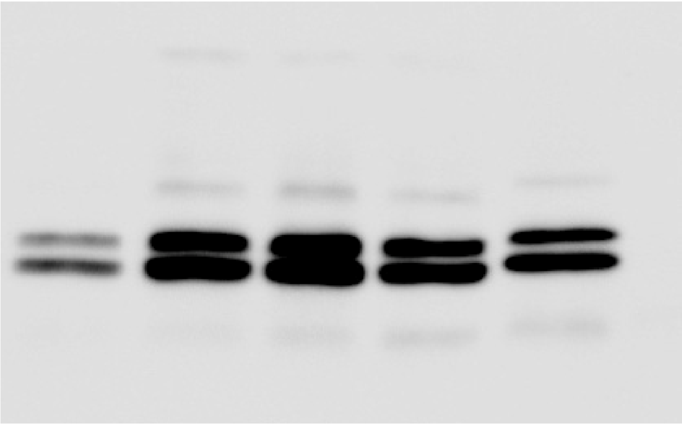

P-ERK

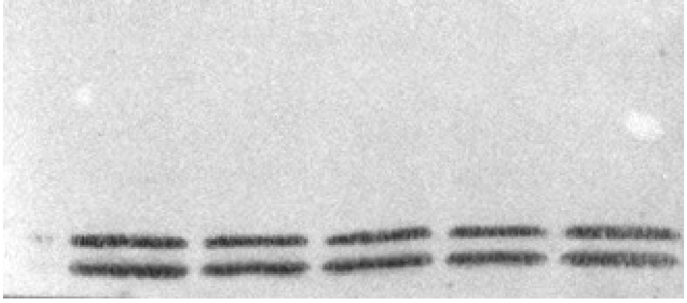

ERK

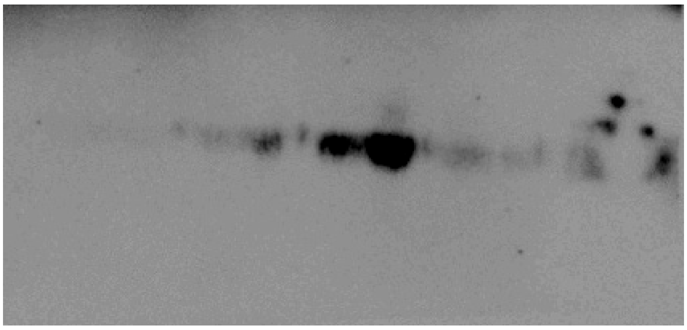

P-IKKα/β

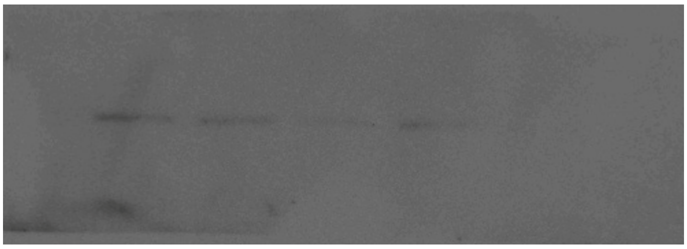

IKKα

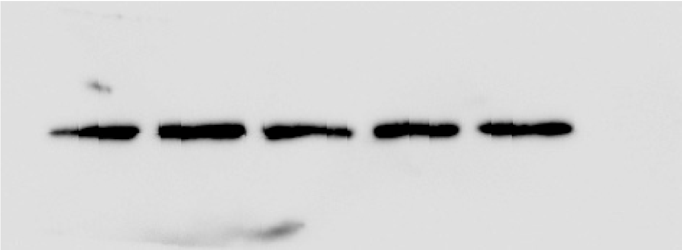

IKKβ

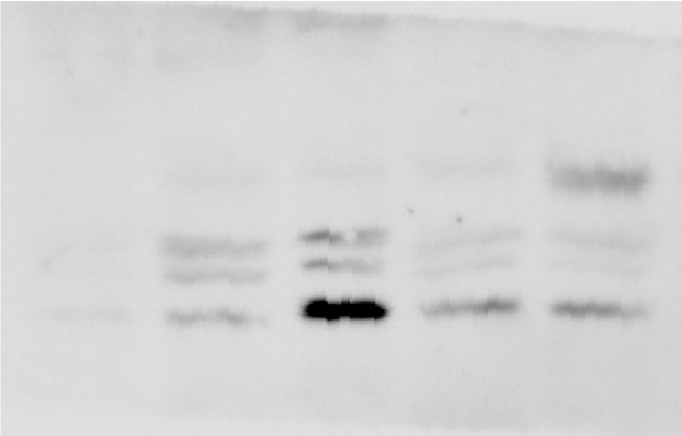

P-IκB

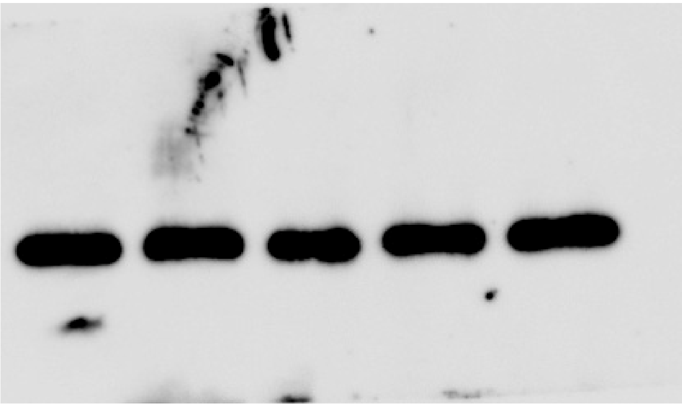

IκB

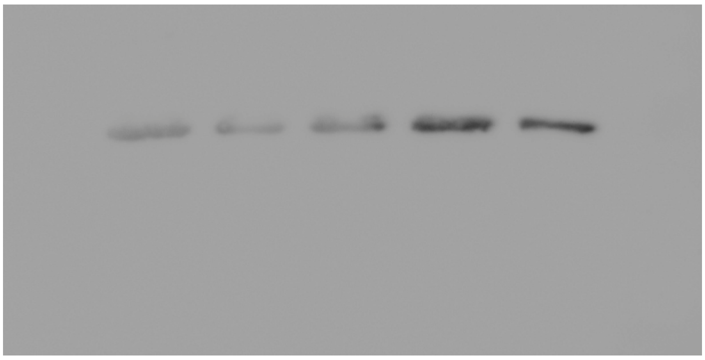

P-NF-κBp65 in cytoplasm

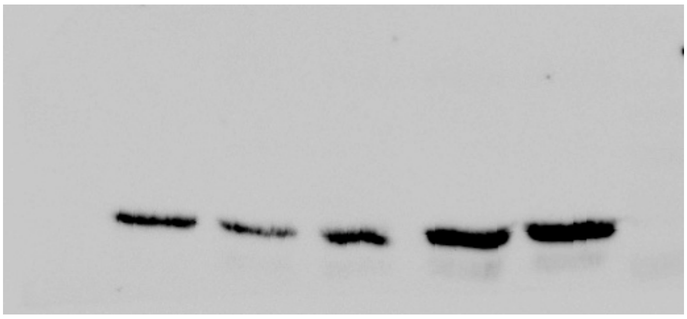

NF-κBp65 in cytoplasm

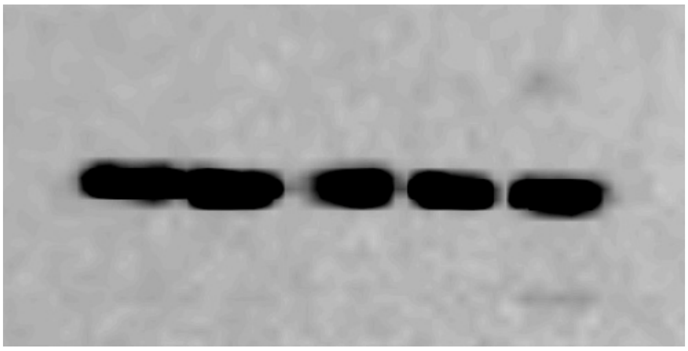

β-actin

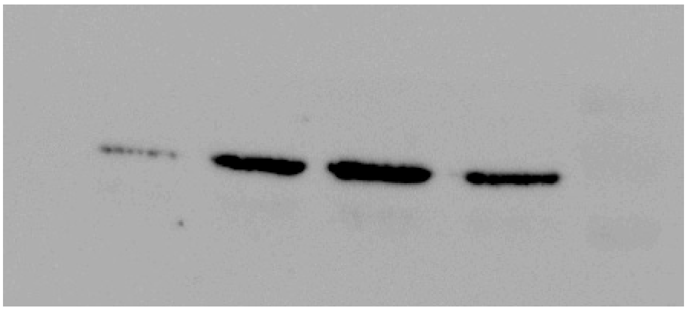

NF-κBp65 in nuclear

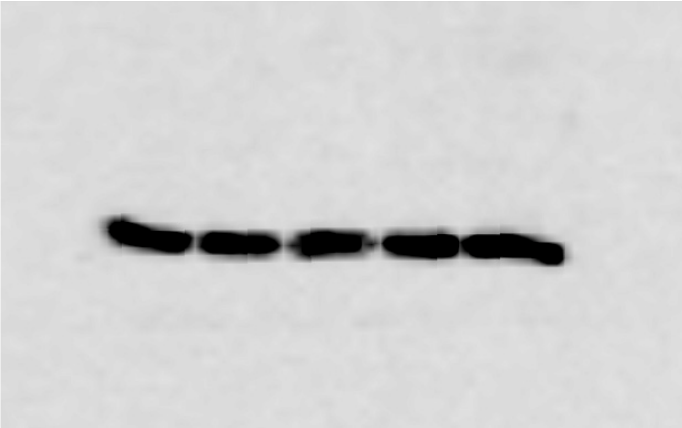

Histone
